# Direct and Indirect Biomimetic Peptide Modification of Alginate: Efficiency, Side Reactions, and Cell Response

**DOI:** 10.3390/ijms22115731

**Published:** 2021-05-27

**Authors:** Anna Golunova, Nadiia Velychkivska, Zuzana Mikšovská, Václav Chochola, Josef Jaroš, Aleš Hampl, Ognen Pop-Georgievski, Vladimír Proks

**Affiliations:** 1 Institute of Macromolecular Chemistry, Czech Academy of Sciences, Heyrovsky Sq. 2, 16206 Prague, Czech Republic; velychkivska@imc.cas.cz (N.V.); miksovska@imc.cas.cz (Z.M.); georgievski@imc.cas.cz (O.P.-G.); proks@imc.cas.cz (V.P.); 2 Department of Histology and Embryology, Faculty of Medicine, Masaryk University, Kamenice 3, 62500 Brno, Czech Republic; chochola.vaclav@gmail.com (V.C.); jaros@med.muni.cz (J.J.); ahampl@med.muni.cz (A.H.); 3 Cell and Tissue Regeneration, International Clinical Research Center, St. Anne’s University Hospital Brno, Pekařská 53, 65691 Brno, Czech Republic

**Keywords:** alginate, adhesion-promoting peptide, polysaccharide modification, cell adhesion, hESC, NMR, XPS

## Abstract

In the fast-developing field of tissue engineering there is a constant demand for new materials as scaffolds for cell seeding, which can better mimic a natural extracellular matrix as well as control cell behavior. Among other materials, polysaccharides are widely used for this purpose. One of the main candidates for scaffold fabrication is alginate. However, it lacks sites for cell adhesion. That is why one of the steps toward the development of suitable scaffolds for cells is the introduction of the biofunctionality to the alginate structure. In this work we focused on bone-sialoprotein derived peptide (TYRAY) conjugation to the molecule of alginate. Here the comparison study on four different approaches of peptide conjugation was performed including traditional and novel modification methods, based on 1-Ethyl-3-(3-dimethylaminopropyl)carbodiimide/*N*-hydroxy succinimide (EDC/NHS), 4-(4,6-dimethoxy-1,3,5-triazine-2-yl)-4-methylmorpholinium chloride (DMTMM), thiol-Michael addition and Cu-catalyzed azide–alkyne cycloaddition reactions. It was shown that the combination of the alginate amidation with the use of and subsequent Cu-catalyzed azide–alkyne cycloaddition led to efficient peptide conjugation, which was proven with both NMR and XPS methods. Moreover, the cell culture experiment proved the positive effect of peptide presence on the adhesion of human embryonic stem cells.

## 1. Introduction

Tissue engineering is a fast-developing area of research, which requires new materials as scaffolds for cell seeding. These materials should not only be biocompatible but also give cells seeded on them mechanical support and provide them a diversity of biological signals to mimic natural tissues.

Due to their intrinsic biocompatibility, natural polysaccharides are now widely used as materials for scaffolds. Among them, alginate is one of the most used for scaffold fabrication. Its ability to form stable hydrogels [1,2] makes it a bio-polymer of choice with high relevance for 3D printing and the development of bioprinting technologies [3] in particular. Nevertheless, the alginate molecule does not contain sites for cell attachment to provide any cell-specific receptor interactions needed for controlling and amending possible cell responses.

One of the ways to tailor alginate’s biofunctionality is the modification with various peptides or other biomimicking moieties [4,5,6,7] via reactions with carboxyl functional groups of polysaccharides. Commonly, fully synthetic short peptide sequences derived from active domains of the extracellular matrix are used for material modification to increase the attraction of cells to the surface and to mediate cell–cell or cell–surface interactions [5]. The selection of the sequences depends on the desired tissue that the scaffold should mimic. One of the most frequently used is RGD peptide derived from fibronectin, IKVAV, and YIGSR peptides derived from laminin [8], HAV and ADT from cadherin [6]. Another promising peptide supporting cell adhesion is TYRAY from bone sialoprotein [9]. This peptide sequence was shown to be effective for human embryonic stem cells (hESC), which have the potential to differentiate into all major cell lineages in the human body [10,11,12]. However, there is a lack of knowledge about the conjugation of TYRAY peptide with alginate.

The conjugation between the molecule of polysaccharide and the peptide goes through the formation of an amide bond. One of the most established ways of amide bond formation is with the use of carbodiimide chemistry. A widely applied method for bioconjugation is zero-length coupling using 1-Ethyl-3-(3-dimethylaminopropyl)carbodiimide/*N*-hydroxy succinimide (EDC/NHS) system [13]. This system was developed for peptide synthesis, where the reaction is usually held between low molecular weight components and often with the use of resin, which altogether provide great availability of the functional groups. However, when used for high molecular weight polymers, the reaction rate is slower due to the lower availability of the functional groups of the polymer caused by the higher viscosity of the solution and steric hindrance. Thus, in this case, the drawbacks of the method can be more distinctive. Among them, a requirement for pH control or the organic solvents environment and the sensitivity of the reaction complex to hydrolysis [13]. Even though, EDC/NHS mediated coupling can also be maintained in water, which is a critical point for the alginate modification, the reaction, in this case, needs acidic pH. In this environment, the amine group on peptide is protonated, which lowers the nucleophilic substitution and leads to lower yields of the reaction [14]. Furthermore, it was shown that this type of coupling may lead to the non-covalent conjugate formation [15].

Triazine-based coupling reagents are another class of zero-length coupling agents [16]. Among them is 4-(4,6-dimethoxy-1,3,5-triazine-2-yl)-4-methylmorpholinium chloride (DMTMM). This coupling agent is water-soluble while being resistant to hydrolysis [17] and, in contrast to the EDC/NHS system, forms stable activated carboxylic acids, which show poor reactivity with alcohols [18]. DMTMM was used for amide condensation [16,17], as well as polysaccharide modification [18,19], including hyaluronic acid [19] and alginate [18,20].

Other ways of conjugating peptides to polymers are click reactions. Among them are the thiol-ene reaction [21,22], strain-promoted azide−alkyne cycloaddition [23], and Cu-catalyzed azide–alkyne cycloaddition (CuAAC) [24]. These approaches need the pre-click functionalization of polysaccharides, but they are promising due to their selectivity. Additionally, the bio-orthogonality of click reactions [25,26] opens many paths for use of the proposed modification approach in the presence of living cells.

In this work, we performed four different traditional and novel modification methods, based on EDC/NHS and DMTMM direct coupling, thiol-Michael Addition, and Cu-catalyzed azide–alkyne cycloaddition reactions, for a peptide to alginate conjugation. We aimed to find the most effective pathway through the direct critical comparison of these methods. Using different approaches, we tried to find the most promising peptide conjugation milieu for further use in the tissue engineering field. Preliminarily, the most promising synthesized conjugate was tested for promoting adhesion in the experiment with human embryonic stem cells (hESCs).

## 2. Results and Discussion

Adjusting biomimetic properties of polysaccharide scaffolds is crucial for tissue engineering. It was shown that the peptide conjugation changes the viscosity of the polysaccharide [27], thus imposing serious obstacles for the development of 3D and bioprinting technologies of bioactive alginates. That is why it is useful to have a mix of: (i) highly modified alginate to secure the cell–material interaction and (ii) neat alginate to secure the viscosity. By adjusting the ratio between these two, the desired molar concentration suitable for cell cultivation can be efficiently achieved [28]. This approach will allow the precise control of the molar concentration in the bioink for 3D printing and thus provide important aspects not only of biofunctionality but also of material processability.

In this research, our goal was to reach 10 mol% of the modification with the peptide. The elemental composition of the alginate before and after the individual modification steps was followed via XPS (Table 1). As XPS is mainly sensitive to the surface of the material, NMR analysis was utilized to gain further information about the chemical structure, yield, degree of substitution (SD), and the efficiency of the conjugation within the bulk structure. However, as the surface composition of the 3D scaffold is of high importance for the successful implementation of the modified alginates, we also perform detailed XPS characterization. The main characteristics of the performed reactions are summarized in Table 2. Obtained conjugates were denoted as follows:

- Alg-TYRAY(EDC) for the direct peptide conjugation with use of EDC/NHS coupling strategy;
- Alg-TYRAY(DMTMM) for the direct peptide conjugation with use of DMTMM coupling strategy;
- Alg-N-AEMI for the N-(2-Aminoethyl)maleimide trifluoroacetate salt (N-AEMI) conjugation DMTMM coupling strategy;
- Alg-PAm for the Propargylamine (PAm) conjugation DMTMM coupling strategy
- Alg-N-AEMI-RGD for the peptide conjugation via thiol-Michael addition;
- Alg-PAm-RGD and Alg-PAm-TYRAY for the peptide conjugation with Cu-catalyzed azide–alkyne cycloaddition.

For further information see the Materials and Methods section.

### 2.1. Alginate Amidation with Help of EDC/NHS

**Figure 1:**
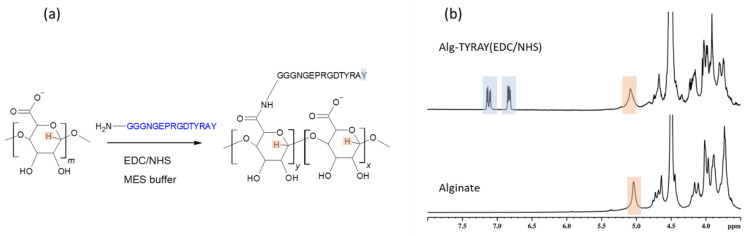
Peptide conjugation to alginate with help of EDC/NHS coupling. (**a**) scheme of the reaction; (**b**) ^1^H NMR spectrum of sodium alginate measured in D_2_O at 50 °C (bottom); ^1^H NMR spectrum of Alg-TYRAY(EDC/NHS) measured in D_2_O at 50 °C (top). Specific signals proving the modification are marked with color.

We reproduced popular modification via carbodiimide chemistry with EDC/NHS zero-length coupling agents (Figure 1a). This is a traditional approach and we used it as a reference to compare with outcomes from more recent modification protocols we describe and employ for the peptide immobilization in further sections. The successful amidation of the alginate with TYRAY peptide led to a rise in the N1s XPS signals to about 8 atomic %. This stems from the established amide bonds between the alginate and the peptide as well as from the amide groups of the TYRAY peptide.

Furthermore, the appearance of characteristic tyrosine signals at the aromatic region (6.7–7.2 ppm) in the NMR spectrum (Figure 1b) proved the success of the modification. However, both the reaction effectivity and the yield were low (Table 1). The use of water buffer solution may lead to an increase in the number of side reactions due to the more favorable reaction of an activated ester with small water molecules instead of bulky peptide molecules. Moreover, low pH may influence the peptide structure through the protonation of amino groups, and thus reducing the nucleophilicity of the conjugate. We note that the main drawback of the EDC/NHS method is in the observed dramatic loss of the peptide. This required us to examine the other coupling protocols (see below).

### 2.2. Alginate Amidation with Help of DMTMM

#### 2.2.1. Peptide Conjugation

**Figure 2:**
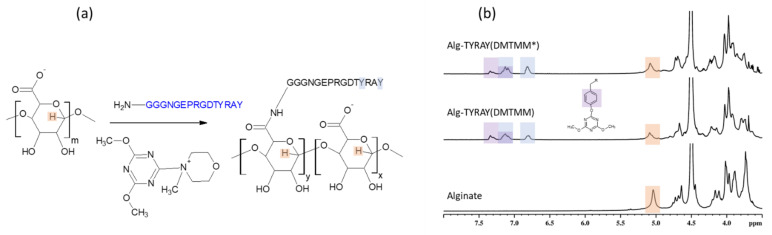
Peptide conjugation to alginate with help of DMTMM coupling. (**a**) Scheme of the reaction; (**b**) ^1^H NMR spectrum of sodium alginate measured in D_2_O at 50°C (bottom); ^1^H NMR spectrum of Alg-TYRAY(DMTMM) measured in D_2_O at 50 °C (middle); ^1^H NMR spectrum of Alg-TYRAY(DMTMM*) measured in D_2_O at 50 °C (top). Specific signals proving the modification are marked with color.

In contrast to traditional coupling reagents DMTMM is known to form stable activated esters in water [29]. Thus, the reaction with this coupling agent (Figure 2a) was expected to have fewer side reactions due to the hydrolysis.

While the XPS results indicated a similar nitrogen content, as in the case of EDC/NHS coupling, the NMR pointed that DMTMM activation method lead to a distinct increase in the substitution effectivity of conjugation (65%) in comparison to the EDC/NHS approach. This points to the same achieved surface concentration of peptide when utilizing EDC/NHS and DMTMM conjugation methods, but a various bulk concentration of peptide within the alginate material.

The Alg-TYRAY(DMTMM) modification was confirmed by the presence of signals at 6.8 and 7.1 ppm (Figure 2b, middle spectrum). Apart from the aforementioned signals, a new deshielded signal at 7.3 ppm was observed. Its presence is probably attributed to 4,6-Dimethoxy-1,3,5-triazine residue from the coupling agent, which may react with the phenol group of tyrosine on the peptide and form a poorly water-soluble 4,6-Dimethoxy-1,3,5-triazine conjugate with tyrosine (Tyr-O-DMT) [19]. DMT residue pulls the electron density from the tyrosine cycle and as a result downfield signal at 7.3 ppm was detected. The ratio between area under the signal at 7.1 ppm (total amount of tyrosine groups) and at 6.8 ppm (non-conjugated tyrosine group) was 0.4 ± 0.1 meaning that almost half of the tyrosine groups were conjugated.

The formation of the non-soluble Tyr-O- DMT adducts explains the observed uncommonly low yield of the reaction (Table 2) as the main weight loss of the water non-soluble product was at the filtration step while purification.

Moreover, to avoid the formation of the Tyr-O-DMT adduct and to achieve a higher yield, we attempted to tailor the procedure. The alginate solution was first stirred with DMTMM (Alg: DMTMM = 1 eq:0.89 eq) for 1.5 h at room temperature, then the solution of the peptide was added. With the lowering of the ratio of DMTMM to alginate it was expected that the coupling agent would be used completely for the carboxyl group activation and would not be left for the side reaction with the peptide. As a result of the modified protocol, we observed an increase in yield (up to 70%). The acquired NMR spectra of the resulted product (Alg-TYRAY(DMTMM)*) showed a decrease of the Tyr-O-DMT adducts concentration 1.8 times. SD was lower than before (5 mol%).

The presence of the side product of the Alg-TYRAY(DMTMM) modification procedure required the development of further approaches.

#### 2.2.2. Functional Amines Conjugation

While traditional conjugation of the peptide to the alginate may face obstacles, DMTMM showed to be an effective coupling agent for polysaccharide amidation, yet only in the absence of phenol group of tyrosine or tyramine [29,30]. As an alternative path, a two-step approach of peptide conjugation was tested. At the first step, a modification of alginate with low molecular amines carrying functional groups suitable for the click reactions was performed, namely N-AEMI for the thiol-Michael Addition and PAm for the CuAAC. For the second step, the conjugation of the peptide of a choice to modified alginate was done.

**Figure 3:**
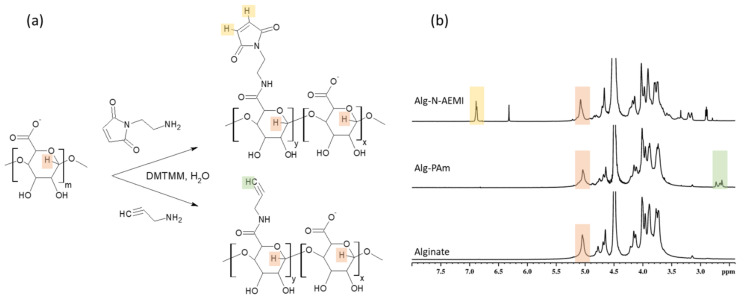
Functional amines conjugation to alginate with help of EDC/NHS coupling. (**a**)-scheme of the reaction; (**b**) ^1^H NMR spectrum of sodium alginate measured in D_2_O at 50 °C (bottom); ^1^H NMR spectrum of Alg-PAm measured in D_2_O at 50 °C (middle); ^1^H NMR spectrum of Alg-N-AEMI measured in D_2_O at 50 °C (top). Specific signals proving the modification are marked with color.

The success of the alginate modification with functional amines (Figure 3a) was proven by XPS and ^1^H NMR spectroscopy. The XPS analysis showed a rise in the N1s signals to about 3.4 and 1.8 at.% for the Alg-N-AEMI and Alg-PAm, respectively. In the NMR spectrum, the resulting Alg-PAm is characterized by the appearance of the triple bond signal found at 2.6–2.7 ppm [30] (Figure 3b, middle spectrum). For the Alg-N-AEMI modification, a new characteristic signal appeared at 7.0 ppm which originates from the hydrogen of maleimide double bond (Figure 3b, top spectrum).

**Figure 4:**
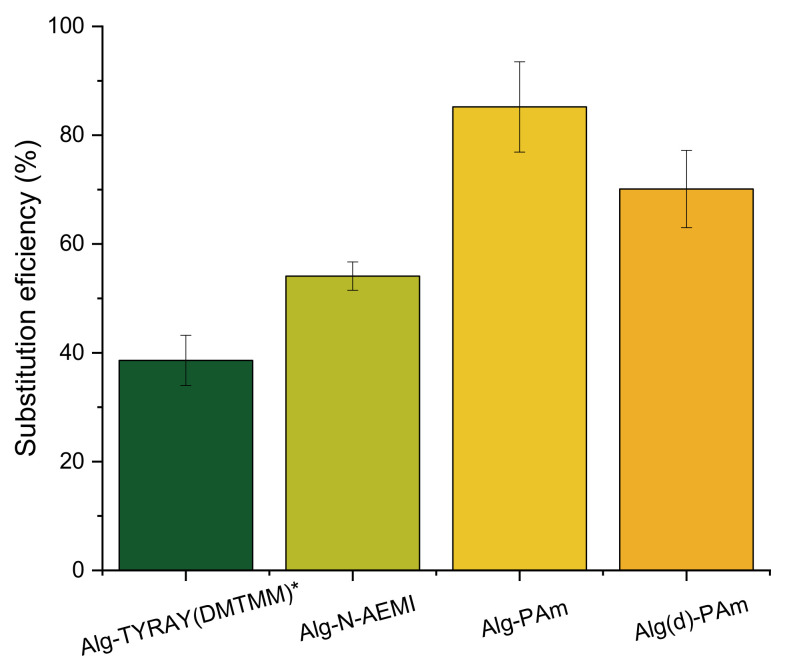
Substitution efficiency of the amidation with DMTMM. All values are reported as mean with the standard deviation (*n* = 3).

As one can see in Table 2, the yields of the reaction with low molecular substitutes were higher than the yield of the reaction with the peptide. The substitution efficiency for N-AEMI was comparable with the peptide reaction when for PAm it was significantly higher (Figure 4). We relate these results to the probable size-dependent diffusion of different molecules towards activated ester groups of alginate when small molecules of functional amines can better penetrate the free volume of alginate coils. Moreover, the solubility (logS) at pH = 7 for PAm is also higher than for N-AEMI (1.93 vs. 1.04 predicted using https://disco.chemaxon.com/calculators/demo/plugins/solubility/, accessed on 20 March 2021), which gives PAm preference in diffusion towards the activated carboxyl groups on alginate.

Yields of the reaction with DMTMM were moderate while the SD was high, meaning that there is a loss of mass during the purification process. We assumed that it might be due to the presence of impurities traces in the pristine alginate. To check this hypothesis, we dialyzed alginate against deionized water first and then conducted the modification with PAm (Alg(d)-PAm). The experiment has shown an increase in the yield of the reaction up to 92% and in the reaction reproducibility. Moreover, the use of purified alginate should increase the accuracy of the reaction load calculation.

### 2.3. Click Reaction

**Figure 5:**
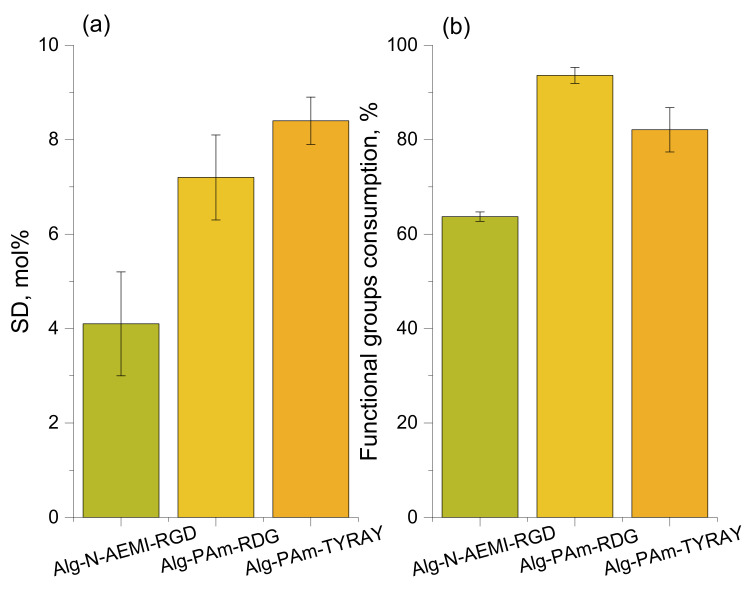
(**a**) The substitution degree for different click reactions; (**b**) the consumption of functional groups on alginate for different click reactions. All values are reported as mean with the standard deviation (*n* = 3).

After a successful functionalization of alginate with functional amines able to undergo click-reactions, we moved to the second step of the two-step modification. Peptides used for click reactions had different functional handles depending on the type of reaction they underwent. For each reaction, the substitution degree and the consumption of functional groups on alginate were calculated (Figure 5).

#### 2.3.1. Thiol-Michael Addition

**Figure 6:**
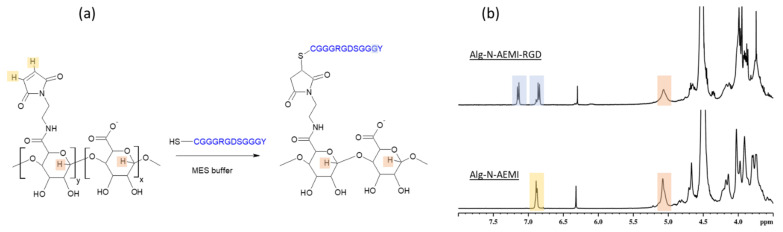
Peptide conjugation to alginate with help of thiol-Michael addition. (**a**) Scheme of the reaction; (**b**) ^1^H NMR spectrum of Alg-N-AEMI measured in D_2_O at 50 °C (bottom); ^1^H NMR spectrum of Alg-N-AEMI-RGD measured in D_2_O at 50 °C (top). Specific signals proving the modification are marked with color.

Alginate modified with N-AEMI (SD = 6 mol%) at the second step of the modification undergone the thiol-Michael Addition reaction between the maleimide group of modified alginate and cysteine of the model RGD peptide (Figure 6a). The success of the conjugation was proven by ^1^H NMR spectroscopy. In Figure 6b the top spectrum shows the presence of the tyrosine signals at (7.1 and 6.9 ppm) along with the vanishing of the signal from the maleimide double bond (bottom spectrum).

**Figure 7:**
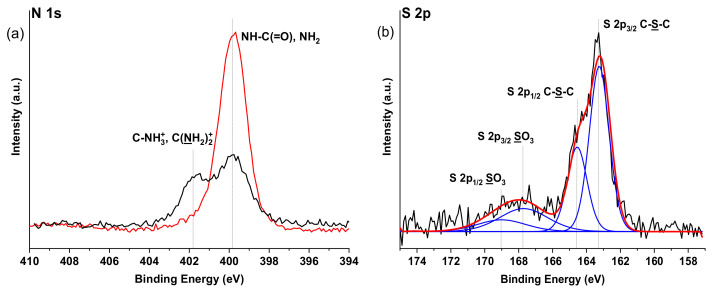
High resolution XPS spectra: (**a**) N1s spectra of Alg-N-AEMI (black) and Alg-N-AEMI-RGD (red); (**b**) high resolution S 2p spectrum shows the successful conjugation of the cysteine group of the peptide to the maleimide group of alginate via the presence of C-S-C moiety.

Also, changes can be seen in XPS spectra (Figure 7) of Alg-N-AEMI-RGD in comparison to Alg-N-AEMI supporting successful peptide conjugation. In Figure 7a the high-resolution N 1s XPS signal can be seen as rich in amide NH-C(=O) originating from peptide conjugated to the modified polymer. In addition to this, we observed a rise of C-S-C signals in the high-resolution S 2p XPS spectrum originating from the formed Michael addition adduct (Figure 7b).

Note, this reaction does not need to be catalyzed [31], which is one of the advantages of this method. Yet there is a possibility of a side reaction between the NH-end of the peptide and the maleimide group. Although it was shown [32] that the thiol conversion in Michael addition is higher when the pH is strongly basic, to hinder the possible conjugation via the terminal amino group of the peptide [31] the buffer with acidic pH should be used. This may lead to the low effectivity of the reaction in used here MES buffer, which reflects obtained data. Only 64% of maleimide groups were substituted resulting in SD of 4 mol% (Figure 5), which was less than for the sample modified with peptide using DMTMM. The coupling yield was similar to that reported elsewhere [9]. The observed low maleimide group consumption can be also connected to the spontaneous formation of disulfides between two cysteine residues of peptides [32]. Thereby it lowers the concentration of the active thiol groups available for the Michael addition.

#### 2.3.2. Cu(I) Catalyzed Azide−Alkyne Cycloaddition

**Figure 8:**
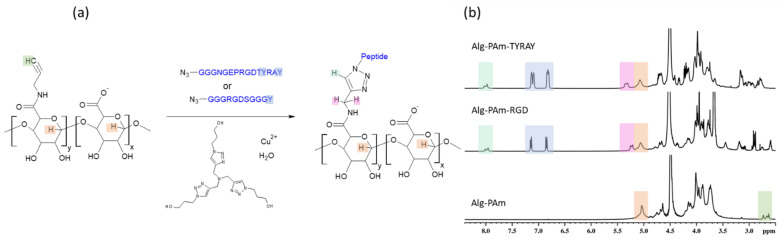
Peptide conjugation to alginate with help of CuAAC. (**a**) scheme of the reaction; (**b**) ^1^H NMR spectrum of Alg-PAm measured in D_2_O at 50 °C (bottom); ^1^H NMR spectrum of Alg-PAm-RGD measured in D_2_O at 50 °C (middle); ^1^H NMR spectrum of Alg-PAm-TYRAY measured in D_2_O at 50 °C (top). Hydrogens assigned to the specific signals are marked with color.

As an alternative to the thiol-Michael Addition, CuAAC reaction between alginate modified with propargylamine and azido-peptide was performed (Figure 8a). The most challenging in this reaction is to stabilize Cu^2+^ ions since they can initiate gelation of alginate [33,34]. For this purpose, we utilized tris(3-hydroxypropyltriazolylmethyl)amine (THPTA) ligand and thus achieved conducting the click reaction without unwanted gel formation. The advantage of THPTA over other chelating agents is in its water solubility. In addition, this ligand showed to speed the CuAAC reaction [35] and protect sensitive peptide moieties from unwanted side reactions [36].

The success of alginate conjugation with both model RGD and target TYRAY peptide was confirmed by ^1^H NMR analysis. One can find tyrosine signals at 7.1 and 6.9 ppm along with the signal from the triazole cycle at 8.0 ppm (Figure 8b). As a result, SD showed good reproducibility with the value of 7.2 mol% for Alg-Pam-RGD and even higher for and Alg-Pam-TYRAY (8.4%). Obtained substitution degrees were higher than for direct peptide coupling with both EDC/NHS and DMMTMM. The propargyl groups consumption was 94% and 82% for Alg-Pam-RGD and Alg-Pam-TYRAY respectfully, which was significantly higher than for the thiol-Michael addition demonstrating the higher effectivity of the CuAAC reaction.

Noteworthy, the aforementioned signal at 8.0 ppm undoubtedly indicates the covalent peptide conjugation and its intensity can be used for the precise SD calculation, which is crucial for the proper characterization of conjugates. Moreover, here, the ratio of intensities of the tyrosine signal to the hydrogen in the triazole cycle (2:1) indicates the absence of the non-covalently bonded peptide.

**Figure 9:**
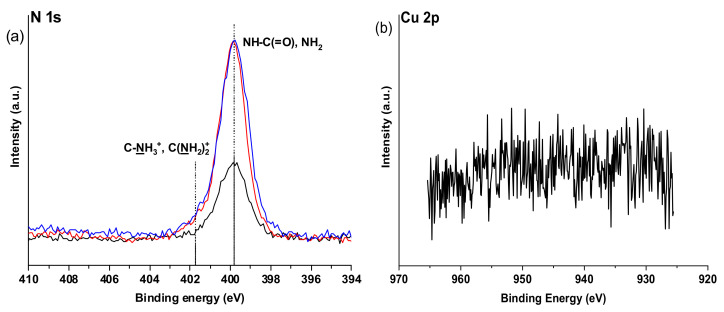
High resolution XPS spectra: (**a**) N1s spectra of Alg-PAm (black), Alg-PAm-RGD (red) and Alg-PAm-TYRAY (blue). The immobilization of the peptides leads to the rise of the amide signals at 399.9 eV and the protonated guanidinium group of arginine at 401.7 eV; (**b**) Representative high-resolution Cu 2p spectrum lacks peaks arising from Cu^2+^ cations.

The results of NMR analysis are in good agreement with XPS data, where one can observe the appearance of strong amide contributions at 399.8 eV in the N 1s spectrum and at the same time the absence of the signal at about 404.5 eV highly characteristic of the free azide group (Figure 9) [37,38]. Additionally, from the XPS spectra, it is possible to assess the increasing amount of nitrogen while the profile of the carbon signals is changing, which indicates conjugation of the nitrogen-rich peptide.

The high-resolution Cu 2p spectra XPS indicated the absence of Cu^2+^ signals in the surface area of the modified alginates. We tentatively assign this to the successful removal of those ions at least in the surface area, however, the absence of Cu^2+^ ions which are also involved in alginate gelation cannot be completely verified. We assume that the modified polymer can be contacted with living cells without fear of their contamination, which is further verified in the cell seeding studies.

The obtained results clearly indicate this approach as the most promising and versatile for biomimetic peptide conjugation among used in this work. Moreover, it allowed us to significantly decrease the loss of the peptides and increase the efficiency of the modification despite the need for pre-click functionalization.

### 2.4. Cell Culture Experiments

**Figure 10:**
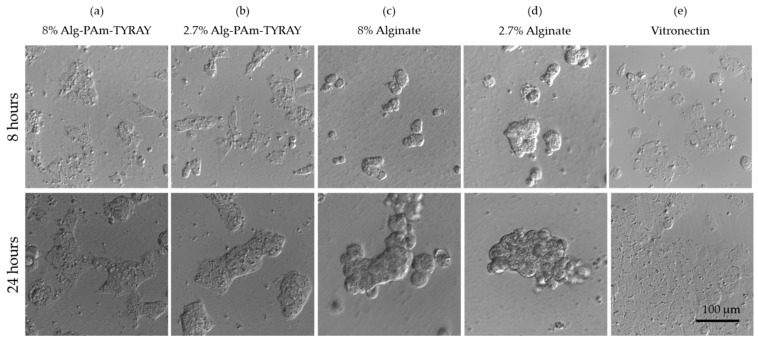
Adhesion and spreading of human embryonic stem cells (hESC) 8 h (top row) and 24 h (bottom row) after seeding on the surface of Alg-Pam-TYRAY (**a**,**b**) and alginate without modification (**c**,**d**). Solutions were prepared in concentrations 8% (*w*/*v*) (**a**,**c**) and 2,7% (*w*/*v*) (**b**,**d**). A positive effect of peptide included in alginates was observed on adhesion and wide spreading of hESCs similar to standard conditions of Vitronectin-coated tissue culture plastic (**e**).

The most promising conjugate (Alg-Pam-TYRAY) was utilized for the preliminary cell culture experiments. These hydrogels are prepared for applications including hydrogel injections and 3D bioprinting, therefore examined hydrogel concentrations were in the range of 2.5–8% which is mostly applied [39,40]. The adhesion and spreading of hESCs on alginate hydrogels were investigated after 8 and 24 h. 8 h after cell seeding, the differences in the cell morphology are evident in the comparison of the modified and non-modified alginates (Figure 10, top row). On the surface of the unmodified alginate hydrogels, hESCs morphology is round-shaped and they soon aggregate (Figure 10c,d). On the contrary, alginates modified with TYRAY allowed hESCs to adhere, spread, and form colonies (Figure 10a,b) in the same dynamics as it was observed in standard cultivation conditions of hESCs seeded on Vitronectin-coated tissue culture plastic (Figure 10e). Similar behavior was observed after 24 h (Figure 10 bottom row) which confirm the stable adhesion and non-toxic properties of hydrogels. Significant differences in cell behavior on hydrogels with different concentrations were not observed.

Even though hESCs are very sensitive to the quality and adhesion properties of the microenvironment, our results show that the modified alginates allow functional cell–material interaction for further control of cell fate.

## 3. Materials and Methods

### 3.1. Materials

Alginic acid sodium salt (180,947), with α-l-guluronic acid (G) to β-d-mannuronic acid (M) (G/M) ratio of 1.56, 4-(4,6-Dimethoxy-1,3,5-triazin-2-yl)-4-methylmorpholinium chloride (DMTMM), N-(2-Aminoethyl)maleimide trifluoroacetate salt (N-AEMI),Propargylamine (PAm), Tris(3-hydroxypropyltriazolylmethyl)amine (THPTA) were purchased from Sigma-Aldrich (Germany). 2-Morpholinoethanesulfonic acid monohydrate (MES) buffer was purchased from Acros Organics (Belgium), N-(3-Dimethylaminopropyl)-N′-ethylcarbodiimide hydrochloride (EDC), *N*-Hydroxysuccinimide (NHS), and reagents for peptide synthesis were from Iris Biotech (Germany). Copper (II) sulfate pentahydrate (CuSO_4_) was purchased from Lach-Ner (Czech Republic). Reagents for cell experiments were purchased from Gibco (Thermo Fisher Scientific, Waltham, MA, USA).

### 3.2. Peptide Synthesis

GGGNGEPRGDTYRAY-NH_2_ (TYRAY), CGGGRGDSGGGY-NH_2_ (RGD), azidoacetic -GGGNGEPRGDTYRAY-NH_2_ (N_3_-TYRAY), and azidoacetic-(PEG)6-GGGRGDSGGGY-NH_2_ (N_3_-RGD) peptides were synthesized by the standard Fmoc/tBu solid-phase method on a TentaGel Rink Amide R resin (0.18 mmol NH_2_/g). An automatic CEM Corporation Liberty Blue microwave peptide synthesizer (Matthews, NC, USA) and software version 1.31.5252.26519 (CEM Corporation, Matthews, NC, USA) were used with default DIIC/OxymaPure coupling and piperidine deprotection cycles. The peptide was cleaved from the resin with a CF_3_COOH/thioanisole/triisopropylsilane/H2O mixture (95/3/1/1 *v*/*v*/*v*/*v*) and isolated by precipitation in diethyl ether. The crude peptide was purified by the high-performance preparative liquid chromatography (Kinetex 5 μm C18 100A, AXIA Packed HPLC column 250 × 21.2 mm), and the identity of the peptide was confirmed using MALDI–TOF analysis.

### 3.3. Alginate Amidation

#### 3.3.1. Peptide Conjugation with the Use of EDC/NHS

Sodium alginate (0.05 M) was dissolved in MES buffer (pH 6.5) overnight. Solutions of the peptide (TYRAY, 0.006 M), EDC (0.06 M), and NHS (0.12 M) in MES buffer were prepared right before the reaction. The final concentration of sodium alginate and peptide in the reaction mixture was 2 *w*/*v*%. First, the solutions of EDC and NHS were added to the alginate solution and left for the activation reaction for 3 h being stirred under ambient conditions. After that, the solution of the peptide was added, and the substitution reaction was maintained for 24 h being stirred under ambient conditions. After the reaction, the mixture was dialyzed (MWCO = 6–8 kDa) against deionized water with an addition of NaCl (2 changes), subsequently with the addition of EtOH (2 changes), and finally with pure water (4 changes). The obtained solution was lyophilized and used for subsequent analysis. Obtained modified alginate (*n* = 3) was noted as Alg-TYRAY(EDC).

#### 3.3.2. Peptide Conjugation with the Use of DMTMM

Sodium alginate (0.025 M) was dissolved in water overnight. Solutions of the peptide (0.003 M) and DMTMM (0.03 M) in water were prepared right before the reaction. Solutions were mixed and left to react for 24 h while stirred under ambient conditions. After that, the reaction mixture was dialyzed as described before. The obtained solution was lyophilized and used for subsequent analysis. Obtained modified alginate (*n* = 3) was noted as Alg-TYRAY(DMTMM).

#### 3.3.3. Functional Amines Conjugation with the Use of DMTMM

Sodium alginate (0.1 M) was dissolved in water overnight. Solutions of low molecular substitutes (N-AEMI or PAm, 0.01 M) and DMTMM (0.1 M) in water were prepared right before the reaction. Solutions were mixed and left to react for 24 h being stirred under ambient conditions. After that, the reaction mixture was dialyzed as described, then lyophilized and used for subsequent analysis and modification. Obtained modified alginates (*n* = 3) were noted as Alg-N-AEMI and Alg-Pam respectfully.

### 3.4. Click-Chemistry

#### 3.4.1. Thiol-Michael Addition

Alginate modified with AEMI (0.05 M) was dissolved in MES buffer overnight. The solution of the cysteine-contained peptide (RGD, 0.005 M) was prepared right before the reaction. Every solution was purged with N_2_ for 15 min. Solutions were mixed and left to react for 24 h being stirred under ambient conditions. After that, the reaction mixture was dialyzed as described before. The obtained solution (*n* = 3) of Alg-NAEMI-RGD was lyophilized and used for subsequent analysis.

#### 3.4.2. Cu(I) Catalyzed Azide−Alkyne Cycloaddition

The click reaction was made according to the protocol [41]. Briefly, the stock solution of Cu-THPTA complex in water (0.01 M) was prepared by mixing CuSO_4_ (0.002 M) solution and THPTA (0.002 M) solution. The stock solution of ascorbic acid (0.005 M) was freshly prepared before the reaction. Typically, alginate modified with propargylamine (final concentration in the reaction mixture 0.025 M) was dissolved in water overnight. The peptide (N_3_-RGD or N_3_-TYRAY) (final concentration in the reaction mixture 0.0025 M) was dissolved in the water right before the reaction. Solutions were mixed along with ascorbate solution (final concentration in the reaction mixture 0.0005 M). The mixture was purged with nitrogen for 15 min and the solution of the Cu-THPTA complex was added (final concentration in the reaction mixture 0.0005 M). The mixture was left to degas for another 15 min and left to react for 24 h being stirred under ambient conditions. The reaction mixture was then dialyzed as described before. The obtained solutions (*n* = 3) of Alg-PAm-RGD or Alg-TYRAY respectfully were lyophilized and used for subsequent analysis.

### 3.5. Analysis

#### 3.5.1. NMR

^1^H NMR spectra were recorded on a Bruker Avance III 600 spectrometer (Billerica, MA, USA) equipped with a 5 mm diffusion probe-head. Measurement conditions were as follows: 90° pulse width 10 μs, acquisition time 5.45 s, spectral width 6002 Hz, relaxation delay 10 s, and 128 scans. The resulting spectra were processed in Topspin 4.1.0, where the integrated intensities were determined with an accuracy of ±1%. During measurements, the stable temperature was maintained within ±0.2 °C using a BVT 3000 temperature unit. Samples for ^1^H NMR analysis were dissolved in D_2_O and measured at 50 °C. Substitution degree was calculated using Equation (1):

(1) SD=ISNH2.56·IG·100%

where *I_S_* is the area under the specific signal, *N_H_* is the number of hydrogen atoms related to the specific signal and *I_G_* is the area under the hydrogen signal at C1 of the guluronic acid (located at 5.1 ppm). Note, that since α-l-guluronic acid (G) to β-d-mannuronic acid (M) (G/M ratio) is 1.56, the intensity of all alginate units (G + M) is 2.56∙*I_G_*.

The efficiency of the reaction was calculated using Equation (2)

(2) $$\mathrm{Efficiency}=\frac{SD}{\mathrm{reaction}\;\mathrm{mixture}\;\mathrm{ratio}\cdot 100}\cdot 100\%$$

where *SD* is the substitution degree calculated using Equation (1), and the reaction mixture ratio is the Alg:Substitute ratio (Table 1).

The modified groups substitution was calculated using Equation (3)

(3) $$\mathrm{Modified}\;\mathrm{groups}\;\mathrm{substitution}=\frac{SD_{2}}{SD_{1}}\cdot 100\%$$

where the *SD*_1_ and *SD*_2_ are the substitution degree of step one and step two of the respected two-step modification process.

#### 3.5.2. X-Ray Photoelectron Spectroscopy (XPS)

XPS measurements were performed using a K-Alpha^+^ XPS spectrometer (Thermo Fisher Scientific, UK) operating at a base pressure of 1.0 × 10^−7^ Pa. The data acquisition and processing were performed using the Thermo Avantage software version 5.9922 (Thermo Fisher Scientific, UK). The dry polymer material was spread on conductive carbon tape and was analyzed using a microfocused (spot size 400 μm), monochromated Al Kα X-ray radiation with a pass energy of 200 eV for the survey and 50 eV for high-energy resolution core level spectra. The X-ray angle of incidence was 30° and the emission angle was along the surface normal. A dual-charge compensation system employing electrons and low energy Ar^+^ ions was employed during the measurements. The analyzer transmission function, Scofield sensitivity factors, and effective attenuation lengths (EALs) for photoelectrons were applied for quantification. EALs were calculated using the standard TPP-2 M formalism. The binding energy scale of the XPS spectrometer was calibrated by the well-known positions of the C 1s C–C and C–H, C–O, and C(=O)–O peaks of polyethylene terephthalate and Cu 2p, Ag 3d, and Au 4f peaks of Cu, Ag, and Au metals, respectively. All measured spectra were charge referenced to the C 1s contribution at a binding energy of 285.0 eV attributed to C–C and C–H moieties.

### 3.6. Cell Experiments

#### 3.6.1. Preparation of Thin Layer of Alginate Gels and Seeding Cells

Unmodified and Alg-Pam-TYRAY modified alginate hydrogels for cell adhesion analysis were prepared by complete dissolution of lyophilized powder in ultrapure water to 0.5% (*w*/*v*) under vigorous stirring and heating (50 °C) overnight, frozen, lyophilized, and gamma-irradiated (200 Gy). Then it was reconstituted in DMEM/F-12 (21,331,020) as an 8% stock solution overnight at 37 °C. Hydrogels were used at a final concentration of 8% and 2.7% (*w*/*v*), diluted in DMEM/F-12.

For thin layers, 75 µL of alginate was pipetted into 24 well plates spread and crosslinked with 100 mM CaCl_2_. Hydrogels were washed twice with DMEM/F-12, human embryonic stem cells were seeded in concentration 75,000 per cm^2^ and cultured in conditioned medium with 5 mM CaCl_2_ in cell culture incubator (5% CO_2_, 37 °C) for 8 and 24 h.

#### 3.6.2. Cell Culture

Undifferentiated human embryonic stem cells, line CCTL 14 ([42]; hPSCreg: MUNIe007-A, RRID: CVCL_C860 at hpscreg.eu (accessed on 01.05.2021)), were grown in feeder-free conditions on tissue culture plastic coated with Vitronectin (A14700, Sigma-Aldrich, Germany) in final concentration 0.5 µg/cm^2^, and using hESC medium conditioned by murine embryonic fibroblasts for 24 h (DMEM/F-12, 15% Knockout serum replacement, 2 mM L-Glutamine, Non-essential amino acids, Penicillin/Streptomycin, 0.1 mM 2-mercaptoethanol (Sigma-Aldrich, Germany), 4 ng/mL human FGF-2 (Peprotech, UK).

After 5 days of growth, hESCs were dissociated by 0.5 mM EDTA into a single-cell suspension and plated at density 75,000 cells cm^2^) on surfaces of alginate hydrogels, as well as vitronectin coated wells, which were used as a positive control for adhesion of hESCs.

## 4. Conclusions

In summary, we performed and critically compared different approaches of one- and two-step peptide conjugation to alginate. We showed the effectivity of DMTMM as a coupling agent for high molecular weight alginate in reactions with different amino group bearing molecules.

The combination of benefits of amidation with DMTMM and click-chemistry approach leads to the highest peptide content in the alginate conjugates among methods used in this work. Using this method, the promising and very efficient pathway of synthesis of a conjugate between alginate and peptides of high bioaplication relevance, such as RGD and TYRAY, was shown and confirmed by both NMR and XPS techniques.

We envisage that this highly effective and reproducible method will be as versatile as for the modification of other polysaccharides with different types of peptides.

## Figures and Tables

**Table 1:** Elemental composition of pristine and modified alginate determined via XPS analysis.

	S 2p	C 1s	N 1s	O 1s	Na 1s
	Atomic %				
Pristine Alg	0.3 ± 0.1	57.6 ± 1.3	1.1 ± 0.2	35.8 ± 0.7	5.3 ± 0.5
Alg-TYRAY(EDC)	1.4 ± 0.4	57.3 ± 1.6	8.3 ± 0.4	31.0 ± 1.6	2.0 ± 0.3
Alg-TYRAY(DMTMM)	0.1 ± 0.1	58.6 ± 1.7	7.0 ± 0.4	32.2 ± 0.7	2.2 ± 0.7
Alg-TYRAY(DMTMM)*^,1^	0.6 ± 0.1	56.6 ± 0.4	7.7 ± 0.9	33.0 ± 1.3	2.1 ± 0.1
Alg-N-AEMI	0.5 ± 0.1	60.1 ± 2.0	3.4 ± 0.3	33.5 ± 1.6	2.5 ± 0.2
Alg-PAm	0.8 ± 0.1	61.9 ± 1.4	1.8 ± 0.1	32.8 ± 1.0	2.7 ± 0.3
Alg-N-AEMI-RGD	0.1 ± 0.1	61.4 ± 4.4	8.6 ± 4.7	27.5 ± 2.1	2.6 ± 1.1
Alg-PAm-RDG ^2^	0.1 ± 0.1	62.3 ± 1.2	5.2 ± 1.2	30.0 ± 1.7	2.4 ± 0.3
Alg-PAm-TYRAY	0.1 ± 0.1	57.3 ± 2.2	7.9 ± 0.6	31.6 ± 1.8	3.1 ± 0.8

^1^ Deferent sequence of steps: Alginate was mixed first with DMTMM and then with peptide solution. ^2^ N.B. We observed no signals of Cu^2+^ in the high resolution Cu2p XPS spectra of the “click” samples.

**Table 2:** Reaction conditions, yields, and substitution degree of the modification for all used methods. The molar ratio between alginate and substitutes was kept at 1 to 0.01 for all the experiments. All values are reported as mean with the standard deviation (*n* = 3).

	Name	Substitute	Reaction Conditions	Yield, %	SD, Mol%
step one	Alg-TYRAY(EDC)	TYRAY	EDC/NHS, MES buffer	36.5 ± 2.7	2.3 ± 1
	Alg-TYRAY(DMTMM)	TYRAY	DMTMM, water	16.7 ± 1.3	7.8 ± 2.4
	Alg-TYRAY(DMTMM)*^,1^	TYRAY	DMTMM*, water	68 ± 2	4.3 ± 0.5
	Alg-N-AEMI	N-AEMI	DMTMM, water	79.8 ± 3.2	6.1 ± 0.3
	Alg-PAm	PAm	DMTMM, water	79.1 ± 7 9	8.6 ± 1.7
	Alg(d)-Pam ^2^	PAm	DMTMM, water	92.7 ± 1.5	7.1 ± 1.4
step two	Alg-N-AEMI-RGD	RGD	MES buffer	36.5 ± 1	4.1 ± 1.1
	Alg-PAm-RDG	N3- RGD	Cu-THPTA, sodium ascorbate, water	72.5 ± 0.6	7.2 ± 0.9
	Alg-PAm-TYRAY	N3- TYRAY	Cu-THPTA, sodium ascorbate, water	74.4 ± 4.5	8.4 ± 0.5

^1^ Deferent sequence of steps: Alginate was mixed first with DMTMM and then with peptide solu-tion. ^2^ Dialyzed alginate was used.

## Data Availability

All data presented in this study are contained within this. Further details are available upon request from the corresponding author.
